# Impact of children with complex chronic conditions on costs in a tertiary referral hospital

**DOI:** 10.11606/s1518-8787.2022056004656

**Published:** 2022-10-07

**Authors:** Regina Maria Antunes Mattiello, Antonio Pazin-Filho, Davi Casale Aragon, Palmira Cupo, Ana Paula de Carvalho Panzeri Carlotti

**Affiliations:** I Universidade de São Paulo Faculdade de Medicina de Ribeirão Preto Departamento de Puericultura e Pediatria Ribeirão Preto SP Brasil Universidade de São Paulo. Faculdade de Medicina de Ribeirão Preto. Departamento de Puericultura e Pediatria. Ribeirão Preto, SP, Brasil; II Universidade de São Paulo Faculdade de Medicina de Ribeirão Preto Departamento de Clínica Médica Ribeirão Preto SP Brasil Universidade de São Paulo. Faculdade de Medicina de Ribeirão Preto. Departamento de Clínica Médica. Ribeirão Preto, SP, Brasil

**Keywords:** Child, Chronic Disease, Tertiary Healthcare, trends, Hospitalization, economics

## Abstract

**OBJECTIVES:**

To investigate the impact of complex chronic conditions on the use of healthcare resources and hospitalization costs in a pediatric ward of a public tertiary referral university hospital in Brazil.

**METHODS:**

This is a longitudinal study with retrospective data collection. Overall, three one-year periods, separated by five-year intervals (2006, 2011, and 2016), were evaluated. Hospital costs were calculated in three systematic samples of 100 patients each, consisting of patients with and without complex chronic conditions in proportion to their participation in the studied year.

**RESULTS:**

Over the studied period, the hospital received 2,372 admissions from 2,172 patients. The proportion of hospitalized patients with complex chronic conditions increased from 13.3% in 2006 to 16.9% in 2016 as a result of a greater proportion of neurologically impaired children, which rose from 6.6% to 11.6% of the total number of patients in the same period. Patients’ complexity also progressively increased, which greatly impacted the use of healthcare resources and costs, increasing by 11.6% from 2006 (R$1,300,879.20) to 2011 (R$1,452,359.71) and 9.4% from 2011 to 2016 (R$1,589,457.95).

**CONCLUSIONS:**

Hospitalizations of pediatric patients with complex chronic conditions increased from 2006 to 2016 in a Brazilian tertiary referral university hospital, associated with an important impact on hospital costs. Policies to reduce these costs in Brazil are greatly needed.

## INTRODUCTION

The reduction in infant mortality rates due to the improvement of social conditions, combined with technological and scientific development, has resulted in the survival of frail children with serious chronic diseases. These children have substantial healthcare needs, including technological dependency, multiple drug dependency, the need for multidisciplinary and home care, functional limitations, and high use of health resources^1^. Despite representing less than 1% of the pediatric population, these children have received increasing attention because of the rising prevalence of their hospital admissions, which greatly impacts health care costs^2^. A North American study showed that chronic complex conditions (CCC) accounted for 8.9% of pediatric hospital admissions and 37.1% of pediatric hospital charges in 1997, whereas, in 2006, the proportion of CCC-associated hospitalizations and costs increased to 10.1% and 40.6%, respectively^3^. In Portugal, the proportion of pediatric patients with CCC admitted to hospitals increased from 14.9% in 2011 to 16.1% in 2015, with a corresponding increase in costs from 38% to 40.8%^4^. In Brazil, two studies reported the hospitalization rates of pediatric patients with CCC and their associated costs. However, no study has showed how these hospitalizations changed over time and their impact on total healthcare costs^5,6^. Considering this knowledge gap and the potential impact of detailing the characteristics and costs related to hospitalization of these children over time, particularly in university hospitals, this study aimed to investigate the impact of CCC on the use of health resources and the costs of hospitalizing pediatric patients in a general pediatric ward of a public tertiary referral university hospital over a decade.

## METHODS

This is a longitudinal study with retrospective data collection and analysis of medical records based on a prospectively constructed database. This study was approved by the institutional research ethics board (CAAE: 19795119.3.0000.5440) and informed consent forms were waived due to its retrospective nature.

This study was conducted in the pediatric ward of a tertiary referral university hospital in the State of São Paulo, which serves a region with an estimated population of 1.5 million inhabitants. At the time this study was conducted, the pediatric ward of the evaluated hospital had 23 beds and admitted children aged one month to 14 years old from its emergency room and pediatric intensive care unit (ICU).

In total, three one-year periods, separated by five-year intervals (2006, 2011, and 2016), were evaluated. All patients who were hospitalized in the studied years were eligible for this study. Demographic, clinical, and outcome data were collected from patients’ health records. For the purposes of demographic analysis, data referring to their first hospitalization were considered. If the same child had been admitted in different studied years, they were considered as a new patient each year.

The presence of CCC was defined at hospital discharge, according to previously published criteria^1,7^. Patients were classified according to the presence or absence of CCC (CCC or non-CCC groups). Moreover, patients with CCC were divided, according to the presence or absence of neurological impairment into CCC-neurological (CCC-NE) and CCC-non neurological (CCC-non-NE) subgroups. Neurological impairment was characterized as static or progressive conditions with involvement of the central nervous (CNS) and/or peripheral nervous systems, resulting in intellectual and/or functional impairment. When a neurological condition was present, patients were classified into the CCC-NE group, regardless of the presence of other CCC. Technological dependency in its most severe degree was considered.

Diagnostic categories were as follows: the CCC-NE group included cerebral palsy (gross motor function classification system - GMFCS - V and < V)^8^, spastic tetraparesis, epilepsy, neurodevelopmental delay, cognitive impairment, hypotonia, neurodegenerative diseases, stroke, and miscellaneous; and the CCC-non-NE group included respiratory, hemato-immunological, gastrointestinal, cardiac, nephrological, orthopedic, oncological, metabolic, and genetic/congenital diseases.

Hospital costs were calculated by applying the absorption costing method to systematic samples of 100 non-CCC and CCC patients (including CCC-NE and CCC-non-NE ones) for each studied year. The absorption costing method includes all (indirect and direct) costs related to the provision of care. Cost centers are identified within the hospital and each one is assigned a previously prorated part of the indirect costs (e.g., electricity, water, security, and cleaning). The total cost of patient care is given by the sum of expenditures in each cost center in which the patient stayed (e.g., operating rooms, intensive care units, and wards), plus direct-cost items (staff, tests, drugs, and consumables)^9^. Patients were systematically sampled according to their rate of participation each year. Each sample of 100 patients consisted of five groups: three with non-CCC patients — in which the most common diseases were associated with hospitalization — and two with CCC patients (CCC-NE and CCC-non-NE). The values obtained in 2006 and 2011 were adjusted by inflation according to the IPCA (broad consumer price index) for December/2016^12^.

### Statistical Analysis

Analysis was made using SAS 9.4 and R 4.0.5. Continuous data were expressed as median (range) and categorical data, as numbers (%). Continuous data between groups were compared by the Mann-Whitney U test and categorical data, by Fisher’s exact test. Two-way analysis of variance was used for cost analysis after the logarithmic transformation of the collected values, due to their great variability. A 5% significance level was considered in all analyses.

## RESULTS

**Figure 1:**
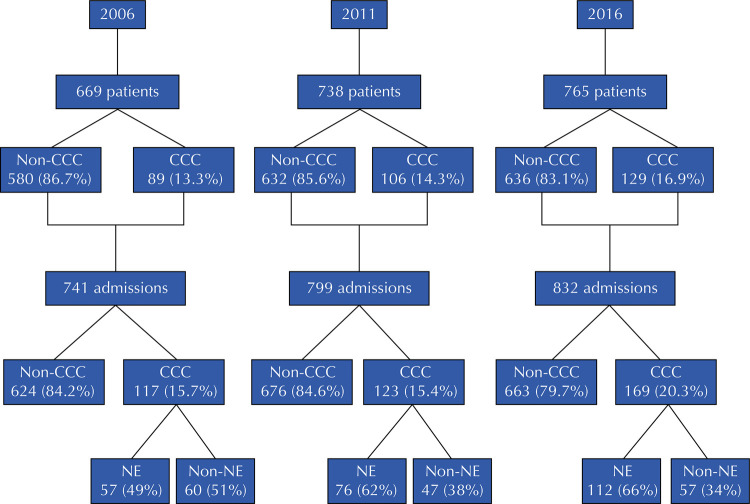
Flow diagram of this study

CCC: chronic complex condition; NE: neurological.

During the studied period, the hospital received 2,372 admissions from 2,172 patients. The proportion of patients with CCC who were admitted to the pediatric ward increased from 13.3% in 2006 to 14.3% in 2011 and to 16.9% in 2016. We also found a rise in the number of hospitalizations of CCC patients over time (117 in 2006, 123 in 2011, and 169 in 2016) (Figure 1). In total, we included 15 patients as duplicates (11 with CCC and four without), six patients whom the hospital had already admitted in 2006 in the 2011 analysis (five with CCC and one without), and nine patients who had had a previous hospitalization in 2011 in the 2016 assessment (six with CCC and three without). If we had excluded them, 13.3%, 13.7%, and 16.2% of inpatients would have shown CCC in 2006, 2011, and 2016, respectively.

Table 1 shows demographic, clinical, and outcome data of CCC and non-CCC patients. In the three studied years, we found a higher proportion of patients aged one to nine years old and a predominance of male individuals in both groups. Multiple hospital admissions and need for ICU were more frequent in patients with CCC, who spent a longer time in the ICU than non-CCC patients. Moreover, in 2011 and 2016, patients with CCC showed approximately three to seven times longer hospital lengths of stay and around five times higher mortality.

**Table 1:** Demographic, clinical, and outcome data of patients with complex chronic conditions (CCC) and without CCC (Non-CCC).

Group	2006		2011		2016	
		
	Non–CCC (n = 580)	CCC (n = 89)	Non–CCC (n = 632)	CCC (n = 106)	Non–CCC (n = 636)	CCC (n = 129)
Age^a^						
< 1 year	97 (16.8)	16 (18)	103 (16.3)	16 (15.1)	58 (9.1)	19 (14.7)
1–9 years	414 (71.6)	68 (76.4)	441 (69.8)	74 (69.8)	427 (67.1)	88 (68.2)
≥ 10 years	67 (11.6)	5 (5.6)	88 (13.9)	16 (15.1)	151 (23.8)	22 (17.1)
Male gender^b^	360 (62.1)	45 (50.6)	378 (59.8)	60 (56.6)	380 (59.7)	76 (58.9)
Admissions/patient^c^						
1	543 (93.6)	70 (78.6)	592 (93.7)	93 (87.7)	610 (95.9)	104 (80.6)
2	30 (5.2)	12 (13.5)	35 (5.5)	11 (10.4)	25 (3.9)	17 (13.2)
≥ 3	7 (1.2)	7 (7.9)	5 (0.8)	2 (1.6)	1 (0.2)	8 (6.2)
Cause of admission						
Trauma	97 (15.5)	6 (5.1)	205 (30.3)	2 (1.6)	267 (40.3)	3 (1.8)
Surgical	74 (11.9)	7 (6)	73 (10.8)	14 (11.4)	127 (19.2)	11 (6.5)
Respiratory	253 (40.5)	60 (51.3)	215 (31.8)	61 (49.6)	107 (16.1)	94 (55.6)
Infection	102 (16.3)	21 (17.9)	82 (12.1)	18 (14.6)	47 (7.1)	15 (8.9)
Other	98 (15.7)	23 (19.7)	101 (14.9)	28 (22.8)	115 (17.3)	46 (27.2)
Need for ICU^d^	46 (7.9)	23 (25.8)	63 (10)	39 (36.8)	46 (7.2)	54 (41.9)
Length of ICU stay (days)^e^	4 (1–17)	7 (1–50)	4 (1–18)	8 (1–52)	5.5 (1–34)	11 (1–196)
Length of hospital stay (days)^f^	6 (0–112)	11 (1–478)	4 (0–100)	11 (1–295)	2 (0–86)	14.5 (1–356)
In-hospital deaths^g^	1 (0.2)	2 (2.2)	0 (0)	6 (5.7)	0 (0)	7 (5.4)

CCC: chronic complex condition; ICU: intensive care unit.

Note: data are expressed as n (%) or median (range). P-values for comparisons between Non-CCC and CCC groups. ^a^ p = 0.23 (2006), p = 0.91 (2011), p = 0.07 (2016). ^b^ p = 0.04 (2006), p = 0.59 (2011), p = 0.92 (2016). ^c^ p < 0.01 (2006 and 2016), p < 0.06 (2011). ^d^ p < 0.01 in the three assessed years. ^e^ p < 0.01 in the three assessed years. ^f^ p = 0.07 (2006), p < 0.01 (2011 and 2016). ^g^ p = 0.10 (2006), p < 0.01 (2011 and 2016).

The progressive increase in the number of patients and hospitalizations of children with CCC was associated with a higher proportion of CCC-NE patient admissions, which rose from 49% to 66% from 2006 to 2016 (Figure 1). Table 2 shows demographic, clinical and outcome data for CCC-NE and CCC-non-NE patients. The demographic profile of both subgroups was similar, with a predominance of children aged one to nine years old. However, the proportion of children under one year of age and those aged 10 years old or above increased over time in the CCC-NE subgroup. The assessed hospital admitted more than three quarters of patients only once. Respiratory diseases were the main cause of hospitalization in both subgroups. However, in the CCC-non-NE subgroup, surgical causes were more frequent than in the CCC-NE subgroup. In the three studied years, technological dependency was higher among patients with CCC-NE, progressively increasing over time in both subgroups. Approximately two thirds of CCC-NE patients had some degree of technological dependency, including the need for enteral or gastrostomy tubes (63.7%), tracheostomy (23%), oxygen supplementation (14.3%), ventriculoperitoneal shunts (10.7%), and mechanical ventilation (7.6%). On the other hand, about 15% of CCC-non-NE patients showed technological dependency, especially the need for enteral or gastrostomy tubes (10.9%) and tracheostomy (5.5%). Associated CCCs were more frequently present in CCC-NE patients than in CCC-non-NE patients in the three studied years (p < 0.0001). We found a progressive increase in the frequency of associated CCCs over time and in the proportion of those affected by two or more associated CCCs in the CCC-NE subgroup. The most commonly associated CCCs in CCC-NE patients were gastrointestinal and respiratory compromise, which affected approximately two thirds and one third of patients, respectively, in the three studied years, whereas 17.5% of CCC-non-NE patients had gastrointestinal impairment in 2016.

**Table 2:** Demographic, clinical, and outcome data of patients with neurological (CCC-NE) and non-neurological (CCC-non-NE) complex chronic conditions.

Subgroup	2006		2011		2016	
		
	CCC–NE (n = 44)	CCC–non–NE (n = 45)	CCC–NE (n = 63)	CCC–non–NE (n = 43)	CCC–NE (n = 89)	CCC–non–NE (n = 40)
Age ^a^						
< 1 year	3 (6.8)	13 (28.9)	6 (9.5)	10 (23.3)	14 (15.7)	5 (12.5)
1–9 years	38 (86.4)	30 (66.7)	46 (73)	28 (65.1)	58 (65.2)	30 (75)
≥ 10 years	3 (6.8)	2 (4.4)	11 (17.5)	5 (11.6)	17 (19.1)	5 (12.5)
Male gender^b^	19 (43.2)	26 (57.8)	37 (58.7)	23 (53.5)	53 (59.6)	23 (57.5)
Admissions/patient^c^						
1	34 (77.3)	36 (80)	53 (84.1)	40 (93)	73 (82)	31 (77.5)
2	8 (18.2)	4 (8.9)	9 (14.3)	2 (4.7)	12 (13.5)	5 (12.5)
≥ 3	2 (4.5)	5 (11.1)	1 (1.6)	1 (2,3)	4 (4.5)	4 (10)
Cause of admission						
Trauma	2 (3.5)	4 (6.7)	1 (1,3)	1 (2.1)	2 (1.8)	1 (1.7)
Surgical	0 (0)	7 (11.7)	3 (3.9)	11 (23.4)	4 (3.6)	7 (12.3)
Respiratory	34 (59.7)	26 (43.3)	45 (59.2)	16 (34)	65 (58)	29 (50.9)
Infection	10 (17.5)	11 (18.3)	12 (15.8)	6 (12.8)	9 (8)	6 (10.5)
Other	11 (19.3)	12 (20)	15 (19.7)	13 (27.7)	32 (28.6)	14 (24.6)
Associated CCC^d^						
0	15 (34.1)	37 (82.2)	18 (28.6)	36 (83.7)	19 (21.3)	26 (65)
1	16 (36.4)	7 (15.6)	22 (34.9)	3 (7)	36 (40.4)	13 (32.5)
≥ 2	13 (29.5)	1 (2.2)	23 (36.5)	4 (9.3)	35 (39.3)	1 (2.5)
Technological dependency^e^	26 (59.1)	6 (13.3)	43 (68.2)	3 (7)	63 (70.1)	9 (22.5)
Need for ICU^f^	11 (25)	12 (26.7)	32 (50.8)	7 (16.3)	41 (46.1)	13 (32.5)
Length of ICU stay (days)^g^	10.5 (2–50)	3 (1–16)	8.5 (1–52)	4(2–33)	14.5 (1–196)	7 (1–31)
Length of hospital stay (days)^h^	13 (2–478)	8 (1–187)	18 (2–295)	8 (1–49)	17 (1–356)	9 (2–207)
In–hospital deaths^i^	1 (1.7)	1 (1.7)	3 (4.8)	3 (7)	6 (7.1)	1 (2.6)

CCC: chronic complex condition; NE: neurological; ICU: intensive care unit.

Note: data are expressed as n (%) or median (range). P-values for comparisons between CCC-NE and CCC-non-NE subgroups. ^a^ p = 0.02 (2006), p = 0.16 (2011), p = 0.60 (2016). ^b^ p = 0.21 (2006), p = 0.69 (2011), p = 0.85 (2016). ^c^ p = 0.27 (2006), p = 0.28 (2011), p = 0.51 (2016). ^d^ p < 0.0001 in the three assessed years. ^e^ p < 0.01 in the three assessed years. ^f^ p = 1.00 (2006), p < 0.01 (2011), p = 0.08 (2016). ^g^ p = 0.03 (2006), p = 0.51 (2011), p = 0.01 (2016). ^h^ p = 0.02 (2006), p < 0.01 (2011 and 2016). ^i^ p = 1.00 (2006), p = 0.67 (2011), p = 0.42 (2016).

The main diagnostic categories of CCC-NE patients were cerebral palsy (n = 99; 50.5%), with almost 75% of the patients affected by the most severe form of the disease (GMFCS V), epilepsy (n = 22, 11.2%), hypotonia (n = 19; 9.7%), and neurodevelopmental delay (n = 17; 8.7%). In the CCC-non-NE subgroup, the most frequent diagnostic categories were hemato-immunological (n = 40; 31.2%), respiratory (n = 26; 20.3%), and gastrointestinal (n = 24; 18.7%) diseases. In total, 70% of patients with hemato-immunological diseases showed diagnoses of sickle cell syndromes; 50% of patients with respiratory diseases, bronchopulmonary dysplasia; and the group with gastrointestinal diseases, the greatest variety (data not shown).

### Cost Analysis

The systematic sample for cost analysis consisted of non-CCC patients hospitalized for appendicitis, musculoskeletal trauma, or respiratory diseases, and CCC patients admitted to the hospital for any reason, according to their hospitalization rates each year (Table 3). As we defined our sample size by the availability of institutional costs section, we compared patients’ technological dependency — which is considered as a marker of severity and of greater use of health resources — to assess if CCC patients’ systematic samples represented the entire population. Results show their samples as comparable to the CCC patient population.

**Table 3:** Systematic sampling of patients for analysis of costs of hospitalization.

Category of patients	2006		2011		2016	
		
	Admissions n (%)	Sample size (n)	Admissions n (%)	Sample size (n)	Admissions n (%)	Sample size (n)
CCC-NE	57 (11)	11	76 (13)	13	112 (18)	18
CCC- non-NE	60 (12)	12	47 (8)	8	57 (9)	9
Non-CCC*	385 (77)	77	443 (79)	79	452 (73)	73
Total CCC-NE + CCC-non-NE + Non-CCC^a^	502 (68)	100	566 (71)	100	621 (75)	100
Total number of admissions/ year	741		799		832	

NE: neurological.

^a^ Patients without complex chronic conditions (CCC) hospitalized for appendicitis, musculoskeletal trauma or respiratory diseases

**Figure 2:**
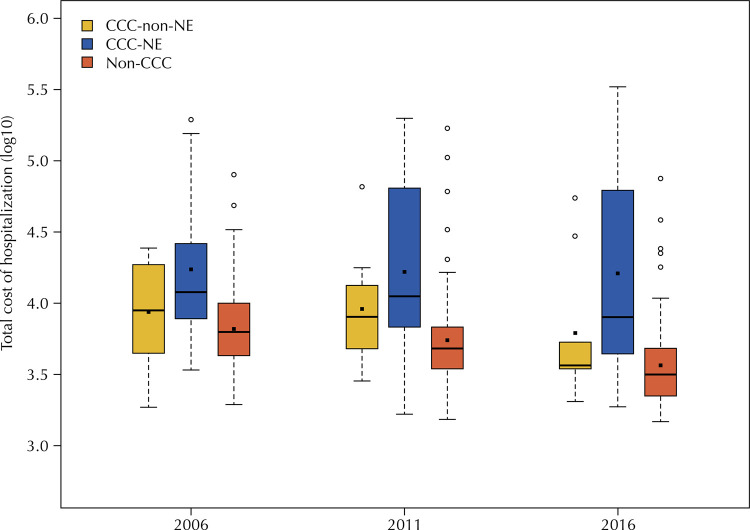
Logarithm of the total cost of hospitalization for patients without complex chronic conditions (Non-CCC) and patients with neurological (CCC-NE) and non-neurological (CCC-non-NE) complex chronic conditions.

CCC: chronic complex condition; NE: neurological.

Note: comparisons between groups: Non-CCC *versus* CCC-NE: p < 0.01 (2006, 2011, and 2016); Non-CCC *versus* CCC-non-NE: p > 0.05 (2006, 2011, and 2016); CCC-non-NE *versus* CCC-NE: p < 0.01 (2016).

In the non-CCC group, hospital costs decreased in 2016, compared to 2006 and 2011 (p < 0.01). Total hospital stay costs were higher for CCC-NE patients than for non-CCC ones in the three studied years (p < 0.01). Moreover, in 2016, total hospital costs were higher for CCC-NE patients than for CCC-non-NE patients (p < 0.01) (Figure 2).

Although hospital costs for CCC-NE patients did not significantly change over time, their impact on hospital bills increased as a result of the higher proportion of CCC-NE patient admissions. In 2006 and 2011, CCC-NE patients accounted for approximately 36% of total costs, whereas, in 2016, their hospitalizations represented 66% of total annual costs. Moreover, total hospitalization costs increased by 11.6% from 2006 to 2011 and by 9.4% from 2011 to 2016 (Table 4).

**Table 4:** Annual hospitalization costs by subgroup in the cost sampling, corrected for inflation to values from December 2016.

	2006		2011		2016	
			
Total annual cost (R$)	1,300,879.20		1,452,359.71		1,589,457.95	
			
Subgroups	Annual cost (R$)	% of total annual cost	Annual cost (R$)	% of total annual cost	Annual cost (R$)	% of total annual cost
Non-CCC	688,232.52	52.9	794,388.96	54.7	423,005.47	26.7
CCC-NE	473,476.07	36.4	535,505.64	36.9	1,055,580.83	66.4
CCC-non-NE	139,170.61	10.7	122,465.10	8.4	110,871.65	6.9

CCC: chronic complex condition; NE: neurological.

## DISCUSSION

This study shows an increase in the number of hospitalized children with CCC from 2006 to 2016, associated with a higher proportion of CCC-NE patients with increasing complexity, as demonstrated by the greater number of associated CCCs, higher technological dependency, and greater use of health resources. We also observed that hospital mortality rose almost 2.5 times from 2006 to 2016 in patients with CCC, an increase possibly related to the progressively greater complexity of these patients. Moreover, the increase in CCC-NE patient admissions contributed to raise total hospitalization costs over time. Our data corroborate the findings of other studies and reinforce the need for reflection on the current care model and its growing impact on hospital costs^4,13^. In our study, we found that 16.9% of admissions to a pediatric ward of a tertiary referral university hospital in 2016 consisted of patients with CCC. The proportion of hospitalized CCC patients reported in the literature varies from approximately 10% in studies involving various types of hospitals to almost 80% in studies carried out in pediatric hospitals or specialized reference centers^3,5,6,13,17^.

The observed increase in hospitalized children with severe neurological impairment over time probably results from regional health system regulation, which refers the most complex cases to tertiary referral university hospitals. This finding is in line with those reported by Berry et al.^18^ in pediatric hospitals in the USA, associated with greater use of resources and higher costs. Gold et al.^19^(2016) highlighted the importance of efficient care after hospital admission to reduce costs, considering that the prevention of hospitalizations is often impossible. Corroborating the importance of this observation, studies have shown great variability in the use of health resources among American pediatric hospitals for similar, specific groups of patients with CCC, reinforcing the need to establish protocols of care for this population to obtain comparable national data^20^. Although the literature on the subject has been advancing rapidly, with an exponential increase in published studies^25^, data from Brazil are still scarce^5,26^. To our knowledge, there is only one Brazilian publication on hospital costs for CCC patients authored by researchers from the National Institute of Women, Children and Adolescents Health Fernandes Figueira (IFF), in Rio de Janeiro. It showed higher costs for CCC patients than non-CCC ones and a positive association between costs and longer hospital stays, number of affected systems, presence of respiratory and metabolic diseases, and use of technologies for diagnosis or treatment^5^. However, although both studies used the same definition of CCC, differences in study design and hospital characteristics preclude comparisons of costs with the results found in this study.

The adaptations needed to face changes in the epidemiological profile within hospitals, associated with the increase in admissions of CCC patients, have been widely discussed, with emphasis in the need for planning, professional training and research to guarantee comprehensive care for these children and their families^27,28^. Indeed, the results of this study highlight the increasing relevance of CCCs in a Brazilian university hospital. Our findings suggest the need for initiatives aimed at greater efficiency in the care of this population, especially in the hospital environment, involving physical and structural adaptations, team training, establishment of quality protocols, support for palliative care teams, and wide integration between hospitalists and specialists, with the creation of care coordination programs. Moreover, it is also important to promote the strengthening of the assistance network of the Brazilian Unified Health System *(Sistema Único de Saúde)* and partnerships between universities for multicenter studies and civil society. Such measures, taken together, can reduce the number of hospitalizations, readmission rates, and length of stay, with consequent reduction in costs^29^.

### Limitations of the Study

A possible selection bias, resulting from the model of care of our hospital, which assists more complex patients referred by the health system regulation, may have hindered the analysis of costs in CCC-non-NE patients. Another limitation is that it was not possible to assess care for all recorded years and cost analysis was limited to samples of 100 patients/studied year. However, we based our study on a prospectively constructed data base with no risk of underreporting or diagnostic errors.

In conclusion, the number of children with CCC admitted to a Brazilian tertiary referral public hospital progressively increased from 2006 to 2016, particularly those with neurological impairment, associated with increased complexity, greater use of health resources, and higher costs. Our data reinforce the need for policies to reduce these costs in Brazil.
